# Psychoactive substance use among outpatients with severe mental illness: A comparative study

**DOI:** 10.4102/sajpsychiatry.v25i0.1111

**Published:** 2019-09-30

**Authors:** Oladipo A. Sowunmi, Gbolagade Amoo, Peter O. Onifade, Adegboyega Ogunwale, Emmanuel Babalola

**Affiliations:** 1 Neuropsychiatric Hospital, Aro, Abeokuta, Nigeria

**Keywords:** psychoactive substance use, drug use, schizophrenia, bipolar disorder, severe mental disorder

## Abstract

**Background:**

Despite several studies on the prevalence and pattern of substance use in Nigeria, there is little information on substance use in patients diagnosed with serious mental illness (SMI) such as schizophrenia and bipolar affective disorder (BD).

**Aim:**

The aim of the study was to compare the pattern of psychoactive substance use among outpatients with BD and schizophrenia.

**Setting:**

The study was conducted in a neuropsychiatric hospital in Nigeria.

**Methods:**

Seventy five consecutive patients with a MINI-PLUS diagnosis of BD were compared with an equal number of patients obtained by systematic random sampling with a MINI-PLUS diagnosis of schizophrenia.

**Results:**

The respondents with schizophrenia were aged 18–59 years (37.2 ± 9.99) and were predominantly young adult (49, 65.3%), men (46, 61.3%), who were never married (38, 50.7%). Overall, lifetime drug use prevalence was 52%, while for current use, overall prevalence was 21.3%. Participants with BD were aged 18–63 years (36.7 ± 10.29) and were predominantly young adult (53, 70.7%), women (44, 58.7%), who were married (32, 42.7%), with tertiary education (31, 41.3%). Overall, lifetime drug use prevalence was 46.7%, while current overall prevalence was 17.3%. These rates (lifetime and current) for both diagnostic groups are higher than what was reported by the World Health Organization in the global status report of 2014 (0% – 16%). The statistically significant difference between the two diagnostic groups was related to their sociodemographic and clinical variables and psychoactive substance use.

**Conclusion:**

Psychoactive substance use remains a burden in the care of patients diagnosed with schizophrenia and BD. Future policies should incorporate routine screening for substance use at the outpatient department with a view to stemming the tide of this menace.

## Introduction

Despite worldwide concern and education about psychoactive substance use, there is limited awareness of the prevalence and pattern of psychoactive substance use among mentally ill patients.^1^ The United Nations reported an increasing trend worldwide in psychoactive substance use with a steady increase in the prevalence of drug abuse and its consequences in Nigeria.^2,3,4^ More than half of the people suffering from schizophrenia or bipolar affective disorder (BD) also present with a lifetime use of psychoactive substances,^5,6,7^ a rate that is much higher than that seen among individuals without bipolar and schizophrenia illnesses.^8^ This phenomenon suggests that, in addition to factors predisposing the general population to psychoactive substance use, there may be factors especially predisposing people with schizophrenia and BD to using psychoactive substances. These additional factors may be related to the reward pathway shown to have abnormalities in both schizophrenia and bipolar disorder patients who are also using substances.^9,10^

Although substance use in both disorders (schizophrenia and BD) makes use of the reward system, the sub-pathways in the reward system may differ in the two disorders. Studies have shown the pathways responsible for psychoactive substance use in schizophrenia and BD but have not indicated which of these are most likely to predispose to psychoactive substance use and abuse.^9^ If the abnormalities in the reward sub-pathway in schizophrenia and bipolar disorder are of a different nature and contribute to the risk of substance use differently in schizophrenia and BD, in addition to genetic, personality and environmental contributions, it may follow that the rates of substance abuse in schizophrenia and BD could be significantly different. From the foregoing, it is imperative to evaluate if this difference exists. Several studies in Nigeria have examined psychoactive substance use among different populations, including students,^11^ hospital-based samples,^12^ communities,^13^ drivers^14^ and prisoners^15^ among others. However, a search through literature showed that few studies have been conducted^7,16,17,18,19,20,21,22^ among people with serious mental illness (SMI) and no published studies were found that compared substance use among patients with schizophrenia and BD in Nigeria. The aim of the study was to compare the pattern of psychoactive substance use among outpatients with BD and schizophrenia in a neuropsychiatric hospital in Nigeria. It is hoped that this study will contribute to the body of knowledge regarding the differences or similarities in the prevalence and pattern of substance use among people with severe mental illness.

## Methods

A cross-sectional comparative study of substance use among patients with the diagnosis of schizophrenia and BD attending the outpatient clinic of the Neuropsychiatric Hospital, Aro, Abeokuta, Ogun State, Nigeria, was carried out between February and March 2015. A systematic random sampling of every fourth patients with schizophrenia was recruited, while consecutive patient with BD were recruited. To be included in the study, patients were between the ages of 18–64 years without chronic and disabling physical conditions (e.g. cerebrovascular disease) or acute medical distress (e.g. acute malaria) and mentally stable to participate in the study. This was determined with the psychotic (clinical judgement section of psychosis M8b, M9b, M10b) and manic (D3c, e, f) modules of the Mini International Neuropsychiatric Interview (MINI PLUS). The sample size was determined using the sample size formula for group comparison according to Whitley and Ball.^23^ Thus, 75 patients with schizophrenia and 75 patients with BD were recruited into this study.

A sociodemographic questionnaire designed by the researcher was used to collect biodata. The MINI PLUS was used in this study to confirm the diagnosis of schizophrenia and BD. The Alcohol, Smoking and Substance Involvement Screening Test (ASSIST) Version 3.1 was used to determine the prevalence and pattern of psychoactive substance use. Data were analysed using the Statistical Package for Social Science (SPSS version 21) computer software. Patients’ occupation was classified according to the system of Boroffka and Olatawura.^24^ The duration of illness was regrouped based on a pattern used in a previous study among outpatients in Nigeria.^25^ Psychoactive substance use in the last 3 months was taken as current use of psychoactive substance in this study. Test for normality was first performed for the age variable using the Kolmogorov–Smirniva test. Subsequently, student *T* test was performed to test the difference in mean ages of patients with schizophrenia and BD. Yates correction was used for Pearson’s chi-square with an expected count less than five (≥ 20%)^26,27^ and multivariate regression analysis was performed to show independent correlation. The level of significance was set at *p* < 0.05 at 95% confidence level.

### Ethical considerations

Ethical clearance was obtained from the Research and Ethics Committee of the Neuropsychiatric Hospital, Aro Abeokuta.

## Results

### Sociodemographic profile of participants

The mean ages of patients with schizophrenia and BD followed a normal distribution.

The mean age of respondents with schizophrenia was not significantly different from that of participants with BD (*t* = 0.29; *p* = 0.77). The only statistically significant sociodemographic variable between the two groups was gender, where men predominated in the schizophrenia group, and women predominated in the bipolar disorder group (χ^2^ = 6.004; *p* = 0.01). This is depicted in Table 1.

**TABLE 1:** Sociodemographic characteristics of respondents (*n* = 150).

Variables	Schizophrenia				Bipolar				Chi-square		
	*n*	%	Age range	s.d.	*n*	%	Age range	s.d.	χ^2^	*df*	*p*
**Age (years)**											
≤ 40	49	65.3	18–59	9.99	53	70.7	18–63	10.29	0.490	1	0.480
< 40	26	34.7			22	29.3					
**Gender**											
Male	46	61.3	-	-	31	41.3	-	-	6.004	1	0.010
Female	29	38.7	-	-	44	58.7	-	-			
**Marital status**											
Never married	38	50.7	-	-	31	41.3	-	-	1.374	2	0.500
Married	26	34.7	-	-	32	42.7	-	-			
Separated or widowed or cohabiting	11	14.7	-	-	12	16.0	-	-			
**Level of education**											
No formal or primary education	11	14.7	-	-	16	21.3	-	-	1.141	2	0.560
Secondary education	31	41.3	-	-	28	37.3	-	-			
Tertiary education	33	44.0	-	-	31	41.3	-	-			
**Tribe**											
Major	69	92.0	-	-	70	96.0	-	-	1.064	1	0.300
Minor†	6	8.0	-	-	5	4.0	-	-			
**Religion**											
Christianity	52	69.3	-	-	50	66.7	-	-	0.123	1	0.726
Islam	23	30.7	-	-	25	33.3	-	-			
**Employment status**											
High socioeconomic	40	53.3	-	-	43	57.3	-	-	0.504	2	0.770
Middle socioeconomic	25	33.3	-	-	21	28.0	-	-			
Low socioeconomic status	10	13.3	-	-	11	14.7	-	-			

s.d., standard deviation.

† , Other tribes in Nigeria aside from Yoruba, Igbo and Hausa.

### Psychoactive substance use among patients with schizophrenia and bipolar affective disorder

The overall lifetime (39, 52%) and overall current (16, 21.3%) pattern of psychoactive substance use among patients with schizophrenia was not statistically different from the overall lifetime (35, 46.7%) and overall current (13, 17.3%) pattern of psychoactive substance use among the BD group and is shown in Table 2. Similarly, there are no significant differences in the prevalence rates of each substance between the two groups of patients.

**TABLE 2:** Comparison of current and lifetime prevalence of psychoactive substance use among patients with schizophrenia (*n* = 75) and bipolar affective disorder (*n* = 75).

Psychoactive substance	Lifetime use							Current use						
	Schizophrenia		Bipolar		χ^2^	*df*	*p*	Schizophrenia		Bipolar		χ^2^	*df*	*p*
	*n*	%	*N*	%				*n*	%	*n*	%			
Any substance	39	52.0	35	46.7	0.427	1	0.51	16	21.3	13	17.3	0.385	1	0.53
Tobacco	15	20.0	10	13.3	1.200	1	0.27	5	6.7	6	8.0	0.118	1	1.00
Alcohol	33	44.0	23	30.7	2.850	1	0.09	11	14.7	10	13.3	0.055	1	0.81
Cannabis	11	14.7	10	13.3	0.055	1	0.81	4	5.3	4	5.3	0.000	1	1.00
Stimulant†	16	21.3	18	24.0	0.152	1	0.69	7	9.3	7	9.3	0.000	1	1.00
Sedative or sleeping pills‡	11	14.7	11	14.7	0.000	1	1.00	3	4.0	2	2.7	0.207	1	0.64
Heroin or morphine or pain medication§	5	6.7	4	5.3	0.118	1	0.73	1	1.3	1	1.3	0.000	1	1.00

† , Ephedrine, coffee and kolanut.

‡ , Lexotan.

§ , Codeine and tramadol.

### Sociodemographic and clinical variables associated with psychoactive substance use among patients with schizophrenia and bipolar affective disorder

The following sociodemographic and clinical variables were statistically significantly associated with lifetime psychoactive substance use in the schizophrenia group: male gender, tertiary education, high socioeconomic status and a single past episode of mental illness. Furthermore, being a young adult was the only statistically significant sociodemographic variable associated with current use of psychoactive substance in the schizophrenia group and no other sociodemographic or clinical variables were statistically significantly associated with current use of psychoactive substance in the schizophrenia group.

Only the following sociodemographic and clinical variables were significantly associated with lifetime use of psychoactive substance in the BD group: male gender, middle socioeconomic status, marital status and duration of illness. In addition, male gender, secondary education, middle socioeconomic status, never married and number of admissions were the only statistically significant sociodemographic and clinical variables associated with current use of psychoactive substance in the BD group, and no other sociodemographic or clinical variables were statistically significantly associated with current use of psychoactive substance in the BD group.

There were no specific types of psychoactive substances used among patients with schizophrenia that demonstrated more than one sociodemographic or clinical variable associated with its use. It was thus unnecessary to subject such associations to multivariate binary logistic regression. Multivariate regression analysis for the BD group is shown in Table 3.

**TABLE 3:** Multivariate regression analysis showing independent correlates of psychoactive substance use among patients with bipolar affective disorder.

Psychoactive substance use	OR	95% CI	*p*
**Lifetime use of any substance**			
**Gender**			
Male	3.23	1.14–9.16	0.027
Female	1.00	-	-
**Duration of illness**			
≤ 6 years	5.63	1.58–20.04	0.008
7–12 years	3.97	1.10–14.29	0.035
≥ 13 years	1.00	-	-
**Lifetime use of alcohol**			
**Gender**			
Male	3.14	0.89–10.99	0.073
Female	1.00	-	-
**Marital status**			
Married	1.00	-	-
Never married	2.38	0.57–9.96	0.236
Separated or widowed or cohabiting	6.13	1.11–33.98	0.038
**Duration of illness**			
≤ 6 years	10.03	1.65–61.18	0.012
7–12 years	8.86	1.45–54.09	0.018
≥ 13 years	1.00	-	-
**Lifetime use of stimulant**			
**Marital status**			
Married	1.00	-	-
Never married	3.44	0.78–15.28	0.104
Separated or widowed or cohabiting	8.44	1.54–46.27	0.014
**Employment status**			
High socioeconomic	1.00	-	-
Middle socioeconomic	3.96	1.08–14.48	0.038
Low socioeconomic	1.83	0.34–9.89	0.482

OR, odds ratio; CI, confidence interval.

## Discussion

The two diagnostic groups were observed to have a similar prevalence of psychoactive substance use, except that in the schizophrenia group cannabis was as prevalent as sedatives or sleeping pills. It should be noted that previous studies in Nigeria did not specifically compare the prevalence rates of these diagnostic groups.

The findings in this study are, however, relatively comparable to previous findings from Nigeria, Europe and the US.^13,28,29,30,31^ However, the minor differences from this study may be related to the population studied; for instance, Gureje’s study was among the general population, the study by Fela-Thomas was conducted in a primary care setting, whereas this study was conducted in a tertiary psychiatric outpatient setting.

Furthermore, different instruments were used; for example, the composite international diagnostic interview was used in Gureje’s study, whereas this study used the MINI PLUS. Consistent with the results from other studies, the use of cocaine, heroin, hallucinogens and inhalants was absent among respondents in this study.^30,31^ A plausible explanation could be that such substances are not affordable when compared to the commoner substances which are cheaper and available to procure.^30^

Gender differences in both diagnostic groups with regard to lifetime and current use of psychoactive substance use were observed in this study. This was similar to earlier studies^19,20,22^ that compared BD and schizophrenia. This observation was reported by Kovasznay, Miller, Ringen and their other coworkers to be related to the fact that male counterparts, may be more explorative than their female counterparts and this may have contributed to our findings. Gender and level of education were significantly related to current use of tobacco in patients with BD. This finding was similar to an earlier study^16^ that also compared schizophrenia and BD. It appears that male gender, never being married and having tertiary education were very important economic determinants in predicting tobacco use in participants with BD.

Male gender and marital status (never married) were associated with lifetime use of alcohol, while male gender, marital status (separated or widowed or cohabiting) and level of education (no formal or primary education) were associated with current use of alcohol in participants with BD. This is similar to other studies^19,20,22^ where a significant relationship was reported in BD as opposed to schizophrenia. The authors of the study speculated that there may be a possible mechanism that predisposes patients with BD to alcohol use, which appears not to be present in patients diagnosed with schizophrenia. This mechanism may be related to the drug reward pathway, which is different in the two diagnostic categories as suggested by the authors. They also indicated that participants with BD tended to show more euphoria and stimulus response to alcohol, which is in keeping with the theory of the brain reward pathway.

This is a plausible explanation for the significant difference observed in this study.

In this study, male gender was an important factor in the lifetime use of cannabis, while marital status (never married) is associated with current use of cannabis in both diagnostic groups. In addition, level of education (secondary) and employment status (middle socioeconomic status) were associated with current use of cannabis in respondents with BD. This is different from reports of previous studies^19,20,22^ where no association was reported.

This may be explained by the relatively smaller sample size of this study compared to the previous study (*n* = 309; 336). In addition, it is suggested that there is a decline in cannabis use with increase in age which may be associated with the effect of cannabis on cognition; however, this is still a subject of debate.^30^

Furthermore, the level of intellectual development if measured with the level of education was a variable determining the use of stimulants in the schizophrenic group, while the financial stability if measured by employment, marital and socioeconomic status was the determining factor for stimulant use in the bipolar group. This is similar to what was reported in a study by Baethge and his coworkers^32^ among patients with schizophrenia and BD. It was noted that stimulant use may be selectively associated with depressive mood in these patients, but those diagnosed with schizophrenia may require some level of cognitive stability to be able to self-medicate, a possible side effect of stimulant use.

On the other hand, the bipolar group, who are relatively cognitively stable, may require large funds to execute this possible self-medication, knowing that extravagance is a symptom of BD. This may explain the difference observed in the diagnostic groups of this study. High socioeconomic status was associated with lifetime use of sedative or sleeping pills in respondents diagnosed with schizophrenia, while sedative or sleeping pills showed no association with the BD group. Fowler, in his study of patterns of current and lifetime use substance use, reported that the use of sedatives or sleeping pills may be associated with dysphoric relief, that is, to relax, and social effect, that is, to beat the boredom and illness and medication-related effects in patients diagnosed with schizophrenia.^33^ This finding may suggest that participants with schizophrenia might have used sedative pills early in the illness to help them get away from or forget the hallucinations they experienced, while respondents with BD may not have seen the need to use sedative pills, especially in the manic phase of the illness. This may explain why an association was observed with schizophrenia participants and not with respondents with BD. In this light, schizophrenia respondents may have used sedatives early at the onset of illness to cope with the symptoms of schizophrenia so as to enable them to function at their place of work.

The age of onset of illness was not significantly related to the lifetime and current use of any psychoactive substance investigated in this study among respondents with a diagnosis of schizophrenia and BD. This is contrary to what has been reported in previous studies,^17,18,34^ where it was reported that substance use was associated with age of onset in both schizophrenia and BD. This difference may be because of methodological differences; for example, the population cohort in the earlier studies included inpatients, who were not investigated in this study, as well as community dwelling respondents. In one of the studies,^35^ where no difference was observed, the sample size was smaller (*n* = 51) than that of this present study, while the study by Kovasznay et al. had a larger sample size and reported an association between duration of illness and psychoactive substance. In Zisook and his coworkers’ submission, they opined that the larger the sample sizes, the more representative of the full spectrum of patients with schizophrenia and affective psychosis the study would reflect.

The association of alcohol use with duration of illness in participants with BD is in keeping with earlier reports that comorbidity of substance use with BD is the most common dual diagnosis.^22,36^ In this study, the number of episodes of illness was observed to be associated with lifetime use of alcohol in respondents with schizophrenia. This was not so in respondents with BD. Alcohol use may also be related to increased rate of metabolism of antipsychotics and mood stabilizers which, in turn, may result in relapse.^36^ It appears that the effect of alcohol on the course of illness is more prominent in the schizophrenia subgroup^18^ and may justify why the association was observed in this study.

The number of admissions was associated with current use of tobacco in both diagnostic groups but only showed association with lifetime use of sedatives in the BD group. BD is a chronic relapsing illness^36^ with high comorbidity with substance use. It is plausible that current use may be associated with the symptom severity, where participants may have used psychoactive substance to ameliorate symptoms related to the illness. In this study, the type of medication used was not significantly associated with psychoactive substance use in both diagnostic groups. This is contrary to what has been reported in earlier studies^37,38^ where a relationship was reported between atypical medication and psychoactive substance use. However, about 80% of both diagnostic groups in this study used typical medication and this may explain the findings in this study.

The finding that male gender was an independent predictor of lifetime use of any substance and lifetime use of alcohol has been reported by several studies both internationally^7,16,19,21,39^ and locally.^30^ This strengthens the suggestion that male counterparts are culturally likely to be permitted to use psychoactive drugs. In addition, because of their risk-taking behaviour male counterparts are more likely to experiment with psychoactive drugs compared to their female counterparts. Duration of illness was also observed to be an independent predictor of lifetime use of any substance and lifetime use of alcohol. This may be related to reports that patients with BD use psychoactive drugs to self-medicate and the impulsivity associated with the illness may worsen over time and contribute to this finding. It was observed that having a dysfunctional family setting (separated or widowed or cohabiting) is an independent predictor of lifetime use of alcohol and stimulants. This was also reported in the epidemiological study conducted by the NASAD team in Nigeria.^12^ This is likely to be so as psychosocial events may serve as precipitants of psychoactive substance use in participant. It may also be seen as a culturally acceptable coping mechanism for people undergoing psychosocial stressors. It was also observed that being of middle socioeconomic status was an independent predictor of lifetime use of stimulants. The finding could be explained by the financial capacity of the respondents involved in this study, and because of the affordability of stimulants, they may serve as a good alternative for participants at the lower end of the social class in this study.^12^

## Limitations

The study was cross-sectional, and therefore, direction of causality could not be established between sociodemographic and clinical variables of patients and psychoactive substance use. The study population involved only one of the many psychiatric hospitals in Nigeria and as such the findings may not be representative of the whole nation, and hence cannot be generalised. The minimum sample size (as calculated) used for this study did not allow for statistical testing for variables with very low prevalence. Also, unstable patients were excluded from this study and this may have influenced the prevalence of psychoactive substance use in both diagnostic groups. However, this study uses an internationally recognised standardised instrument in assessing psychoactive substance use among patients with major mental illness, which is a strength of the study, and allows for replicability in other settings.

## Conclusion and recommendations

Alcohol was the most prevalent psychoactive substance used by participants with SMI in this study and age group less than 40 is associated with current substance use in participants with schizophrenia, while male gender, secondary education, middle socioeconomic status, marital status and duration of illness were associated with current substance use in participants with BD. Therefore, it is recommended that selective prevention strategies for psychoactive substances should target people with SMI who have the above sociodemographic and clinical variables. Cutting off, cutting down or prevention of substance use from progressing to misuse disorder will have direct and indirect effect on the course of the illness, effectiveness of medication used in treatment and the prognosis. There is a need to increase and provide screening instruments for the use of alcohol and other psychoactive substances. It is also important to educate patients regularly on the negative consequences of psychoactive substance use and its effect on treatment and outcomes. The consequences of psychoactive substance use on the course of schizophrenia and BD can be mitigated by brief drug interventions that can be administered easily by medical practitioners. Medical personnel should be trained in the use of ASSIST, which will help to detect early substance use problems in order to institute early intervention. Future studies could focus on possible associations with other clinical measures such as motivation for use, impulsivity and cognitive decline, as well as biological parameters such as genetics and brain imaging. Finally, a longitudinal study may be helpful in showing direction of causality.
